# P300 and delay-discounting in obsessive–compulsive disorder

**DOI:** 10.1007/s00406-021-01302-7

**Published:** 2021-07-13

**Authors:** Vera Flasbeck, Björn Enzi, Christina Andreou, Georg Juckel, Paraskevi Mavrogiorgou

**Affiliations:** 1 Department of Psychiatry, LWL-University Hospital, Ruhr University Bochum, Alexandrinenstr. 1, 44791 Bochum, Germany; 2 Department of Psychiatry and Psychotherapy, University Hospital Lübeck (UKSH), Ratzeburger Allee 160, 23538 Lübeck, Germany

**Keywords:** OCD, Event-related potentials, P300, Delay discounting, Neuroimaging, fMRI

## Abstract

Previous research showed that dysfunctions of fronto-striatal neural networks are implicated in the pathophysiology of obsessive–compulsive disorder (OCD). Accordingly, patients with OCD showed altered performances during decision-making tasks. As P300, evoked by oddball paradigms, is suggested to be related to attentional and cognitive processes and generated in the medial temporal lobe and orbitofrontal and cingulate cortices, it is of special interest in OCD research. Therefore, this study aimed to investigate P300 in OCD and its associations with brain activity during decision-making: P300, evoked by an auditory oddball paradigm, was analysed in 19 OCD patients and 19 healthy controls regarding peak latency, amplitude and source density power in parietal cortex areas by sLORETA. Afterwards, using a fMRI paradigm, Blood–oxygen-level-dependent (BOLD) contrast imaging was conducted during a delay-discounting paradigm. We hypothesised differences between groups regarding P300 characteristics and associations with frontal activity during delay-discounting. The P300 did not differ between groups, however, the P300 latency over the P4 electrode correlated negatively with the NEO-FFI score openness to experience in patients with OCD. In healthy controls, P300 source density power correlated with activity in frontal regions when processing rewards, a finding which was absent in OCD patients. To conclude, associations of P300 with frontal brain activation during delay-discounting were found, suggesting a contribution of attentional or context updating processes. Since this association was absent in patients with OCD, the findings could be interpreted as being indeed related to dysfunctions of fronto-striatal neural networks in patients with OCD.

## Introduction

Obsessive–compulsive disorder (OCD) is a psychiatric condition that involves neurobiological dysfunctions of fronto-striatal neural networks. Neuroimaging methods have contributed to a better understanding of the pathogenesis of this disorder, however, findings are not consistent across all studies. Although efficacious treatments have been developed and established, patients in clinical settings often show inadequate responses to treatment attempts. Several studies indicate a neurobiological basis of OCD, resulting in two main hypotheses: neuroanatomical and serotonergic. Studies using neurochemical and neuroimaging methods have shown that various neurotransmitters are implicated in the pathophysiology of this disorder, including serotonin [1], dopamine [2] and glutamate [3]. To date, the highest impact is attributed to the neurochemical model of OCD that postulates a central serotonergic dysfunction, mainly based on the efficacy of selective serotonin reuptake inhibitor (SSRI) treatment in OCD. However, the underlying therapeutic mechanism of SSRIs in OCD remains unclear because there are discrepant findings across studies of structural and functional brain changes before and after SSRI treatment in patients with OCD [4].

In addition, it has been suggested that OCD is caused by abnormal activity in the cortico-striato-thalamo-cortical (CSTC) circuits, including the orbitofrontal cortex (OFC), the striatum within basal ganglia and the thalamus [5, 6], which is summarised as the neuroanatomical hypothesis. It was postulated that OCD symptoms may be related to increased activity in the OFC, as a consequence of diminished inhibitory effects of the striatum (especially the globus pallidus internus) on the thalamus. Furthermore, this hypothesis suggests that OCD could be associated with dysfunctional cognitive and metacognitive processing. In order to investigate the proposed OFC hyperactivity in OCD patients, the P300 component of auditory event-related potentials (ERPs) could be a suitable tool, as it is proposed that P300 is generated in the medial temporal lobe, OFC and cingulate cortex [7]. Furthermore, the appearance of P300 during oddball paradigms is suggested to reflect cognitive and attentional processes. In detail, the P300 occurs with a latency of approximately 300–500 ms after the occurrence of rare or task-related stimuli or after a target stimulus (compared to non-target stimuli) and was measured over frontal-to-temporal and parietal electrodes.

A tremendous range of literature revealed inconsistent cognitive neuropsychological findings e.g. attentional deficits in OCD, which were found using various behavioural tests. The investigation of biological markers, such as the P300 component, also contributed to the understanding of cognitive alterations in OCD. Unfortunately, inconsistent P300 abnormalities were reported for patients with OCD with several previous studies reporting shortened latencies [8–12] and increased amplitudes [8, 13–15], whereas other studies showed decreased P300 amplitudes in these patients [16, 17]. Thus, additional future research is necessary to clarify the P300 alterations and in OCD, which was one aim of the present study.

Further studies aimed to investigate ERPs, especially the P300, and their changes during decision-making tasks [18–22]. Here, the P300 was found to be linked to risky decision making, with larger P300 amplitudes associated with riskier behaviours. Besides these findings, only a few studies exist that were interested in specific delay-discounting effects on P300, such as the effect of intertemporal choices [23–26].

Research regarding decision making in difficult tasks, such as the Iowa Gambling Task [27] and the Game of Dice Task [28], also showed abnormal performances in OCD patients [29–32], but did not clarify which neural processes were altered. Delayed reward discounting is a behavioural economic index of impulsivity and numerous studies have examined delayed reward discounting in substance use disorder [33, 34]. However, few empirical data is available on delayed reward discounting in patients with OCD [35]. In a series of functional magnetic resonance imaging (fMRI) studies, scientists reported activities primarily within the OFC during delay-discounting tasks [36], therefore, this task may be a suitable tool for assessing the activity state within the OFC. However, it remains unclear whether neurotransmitters, especially serotonin, are involved in the abnormalities of the CSTC circuit in OCD. In a recently published study, using the same dataset as the present investigation, the results indicated that activation of dorsolateral and medial prefrontal cortex (PFC) as well as ventral striatum activation differed between OCD patients and healthy volunteers during the delay-discounting paradigm (immediate reward vs. control) [37]. Based on previous literature and theoretical considerations, we propose that P300, as a marker of cognitive and attentional processes, would be increased in OCD, due to altered attention and accelerated cognitive and motor processes. Higher P300 processing would be observable as lower amplitudes and longer latencies [38]. Moreover, it would be of interest to examine whether general cognitive processing would be associated specifically with OFC activity in patients with OCD. The OFC activity would be of special interest in OCD since it is suggested to be a region which is functionally altered in OCD, during a task that is known to elicit deviating behaviour in OCD patients compared und unaffected individuals.

To our knowledge, the approach of combining oddball P300 measures with BOLD contrasts of delay-discounting has not been investigated previously and is the secondary aim of the present investigation. We combined the previously performed fMRI analysis during the delayed discounting paradigm with EEG, cortical and source analysis concerning the P300 component, whereby these measurements were conducted consecutively. For the fMRI analysis, functional BOLD signal was extracted from selected anatomically defined regions of interest in the OFC, next to whole brain fMRI analysis [37].

We hypothesised that a significant association of P300 during EEG recording would be found with activation of the reward processing system during the fMRI-delay-discounting task. Here, we proposed that a higher cognitive demand during the P300 paradigm would be related to increased OFC activity. In addition, we expected to detect differences in P300 amplitude and latency between healthy participants and patients with OCD. More detailed, we hypothesised to find longer latencies and decreased amplitudes in patients with OCD, since we suggested increased cognitive impairment in these patients.

## Method

### Subjects

Nineteen patients (eight females; mean age 33.37 ± 11.73 years) with unequivocal diagnosis of OCD were recruited. Diagnosis was based on the diagnostic criteria of the 4th edition of the *Diagnostic and Statistical Manual of Mental Disorders* (DSM-IV) [39] and 10th revision of the *International Statistical Classification of Diseases and Related Health Disorders* (ICD-10: F42.X) [40]. Exclusion criteria included organic disorders according to the ICD-10 (F0X) or recent concomitant neurological or other medical disorders and the presence of severe alcohol or substance abuse. No patient met the criteria for Tourette syndrome or any psychotic disorder. Table 1 shows the sociodemographic and clinical data of the nineteen patients included in the study. Seventeen patients were medicated at the time of assessment: Thirteen were taking SSRIs (fluoxetine, 40–60 mg/day; sertraline, 50–150 mg/day; escitalopram, 10 mg/day; citalopram, 20–60 mg/day), one received clomipramine (200 mg/day) and three received a serotonin–norepinephrine reuptake inhibitor (SNRI: venlafaxine, 300 mg/day, *n* = 2; or duloxetine, 90 mg/day, *n* = 1). None of the patients were engaged in cognitive-behavioural therapy during the study period.

**Table 1:** Sociodemographic and clinical characteristics of patients with obsessive–compulsive disorder (OCD) and healthy controls

	OCD (*n* = 19)	Controls (*n* = 19)
Gender		
Female	8(42.1%)	8 (42.1%)
Male	11(57.9%)	11 (57.9%)
Age (years)	33.37 ± 11.73	31.63 ± 10.79
Marital status		
Married	3 (15.8%)	4 (21.1%)
Cohabitating	10 (52.6%)	8 (42.1%)
Single	6 (31.6%)	7 (36.8%)
Education		
Upper grade	15 (78.9%)	16 (84.2%)
Middle grade	4 (21.1%)	3 (15.8%)
Lower grade	0	0
Occupational status		
Employed	8 (42.1%)	13 (68.4%)
Unemployed	3 (15.8%)	0
Student	6 (31.6%)	6 (31.6%)
Retired, unable to work	2 (10.2%)	0
Duration of illness (years)	14.27 ± 12.39	
Age of onset (years)	19.21 ± 6.71	
HAM-D	12.42 ± 6.13	
BDI	14.68 ± 10.12	1.42 ± 2.01*
Y-BOCS, obsessions	10.74 ± 2.53	
Y-BOCS, compulsions	10.53 ± 3.73	
Y-BOCS, total	21.79 ± 6.59	
MOCI	14.84 ± 5.93	3.89 ± 2.96*
STAI I	42.89 ± 13.72	30.21 ± 5.06*
STAI II	50.26 ± 11.75	30.58 ± 7.95*
CGI	4.58 ± 0.69	1.00 ± 0*
MWST-IQ	109.63 ± 12.08	119.58 ± 13.22*
NEO-FFI, total	2.77 ± 0.55	2.69 ± 0.69
BIS-11, total	59.00 ± 8.72	56.37 ± 7.43
PSP	67.16 ± 14.08	100*

Values are numbers and percentages or means and standard deviations (SD); *p < 0.05

*HAM-D* Hamilton Depression Scale, *BDI* Beck Depression Inventory, *Y-BOCS* Yale–Brown Obsessive Compulsive Scale, *MOCI* Maudsley Obsessive–Compulsive Inventory, *STAI* Stait–Trait Anxiety Inventory, *CGI* Clinical Global Impression scale, *MWST-IQ* Mehrfach-Wortschatztest, *NEO-FFI* NEO Five-Factor Inventory, *BIS-11* Barratt Impulsiveness Scale, *PSP* Personal and Social Performance scale

Nineteen healthy volunteers (eight females; mean age 31.63 ± 10.79 years) without any neurological or psychiatric disorder in their personal or family history served as a control group, matched for age, gender, education level and handedness (18 right-handed). The volunteers underwent the Mini International Neuropsychiatric Interview for DSM-IV and ICD-10 disorders (MINI-PLUS) [41, 42] and psychometric tests for obsessive–compulsive, depressive and anxiety symptoms.

All participants underwent the same study design with fMRI, P300-based electroencephalography (EEG) and psychometric assessments within a few hours on a single day. All participants started with the EEG recording and questionnaires in the morning and the fMRI recording was done in the afternoon. For one control participant, the fMRI recording was done the next morning, still within 24 h.

### Clinical assessment

The severity of OCD symptoms was assessed by the Yale–Brown Obsessive Compulsive Scale (Y-BOCS) [43, 44] and the Maudsley Obsessive–Compulsive Inventory (MOCI) [45]. To validate the presence of OCD symptoms, we used the Y-BOCS symptom checklist.

The severity of depressive symptoms was assessed using the Hamilton Depression Rating Scale (HAM-D) [46] and self-ratings were assessed by the Beck Depression Inventory (BDI) [47]. Anxiety symptoms were measured using the State-Trait Anxiety Inventory (STAI I and II) [48, 49]. The overall severity of the psychiatric disorder was quantified using the Clinical Global Impression (CGI) score (NIMH) [50]. Psychosocial functioning was measured by the Personal and Social Performance scale (PSP) [51] and impulsivity was assessed by the Barratt Impulsiveness Scale (BIS-11) [52, 53]. The NEO Five-Factor Inventory (NEO-FFI) [54] was used to assess personality characteristics such as extraversion, neuroticism and conscientiousness. Participants’ verbal intelligence was estimated with the Mehrfachwahl–Wortschatztest (MWT) [55].

### P300

During the oddball paradigm, two different kinds of stimuli (80% non-target, 400 sinus tones, 500 Hz; 20% target stimuli, 100 sinus tones, 1000 Hz) were presented in pseudorandomized order (80 dB SPL, 40 ms duration, 10 ms rise and fall time, interstimulus interval 1.5 s) via headphones (Sony Stereo Headphones MDR-1A, Sony^®^ Corporation) and Presentation® software (Neurobehavioral Systems, Inc., Version 14.9, Berkeley, CA: www.neurobs.com) to the participants. All participants were instructed to press a response button with their dominant hand whenever they heard the target stimulus.

### EEG recording and data analysis

Subjects sat in a comfortable armchair in an electrically shielded and sound-attenuated room. Auditory-evoked potentials were recorded with 32 non-polarizable Ag–AgCl electrodes referred to as FCz, placed according to the international 10/20 system. Impedances were kept at 5 kΏ or below. EEG was filtered using a bandpass of 0.16–70 Hz and data were collected at a sampling rate of 250 Hz using a BrainAmp MR amplifier and BrainVision recorder software (Version 1.20.001: Brain Products GmbH, Gilching, Germany). Data analysis was performed using the BrainVision Analyzer 2.0 (Version 2.01.3931: Brain Products GmbH, Gilching, Germany). The recorded data were re-referenced to the mastoid electrodes and filtered using bandpass and notch filters (0.5–20 Hz and 50 Hz). For artifact rejection, all trials were excluded if the voltage exceeded ± 70 µV in any channel. The epochs (− 200 to 1000 ms) were averaged separately for the target and non-target stimuli and corrected to the baseline (− 200 ms). Only subjects with at least 40 trials free of artefacts for both stimuli were included.

**Fig. 1:**
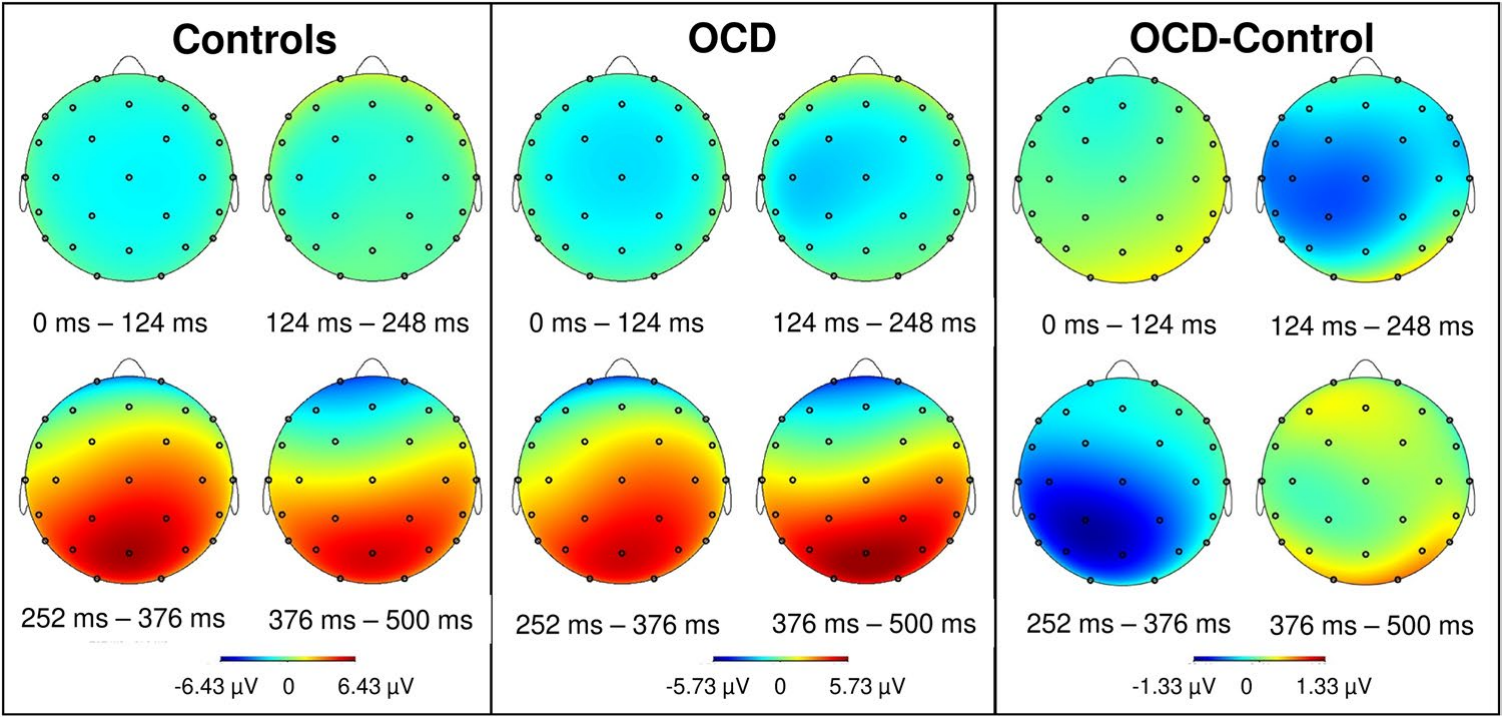
Topographic maps of brain activity after onset of the target tones from 0 to 500 ms in healthy controls (left) and patients with OCD (middle), measured by EEG. The right topographic maps show the difference between patients with OCD and healthy controls

The P300 amplitudes and peak latencies were analysed (P300 defined as the most positive peak within 250–500 ms after stimuli onset for the P3, P4 and Pz electrodes because P300 is suggested to be maximal over parietal electrodes [56]. This was also true for the present study. As shown in Fig. 1, the maximal amplitude was recorded over parietal electrodes, independent of group.

### sLORETA analysis

**Fig. 2:**
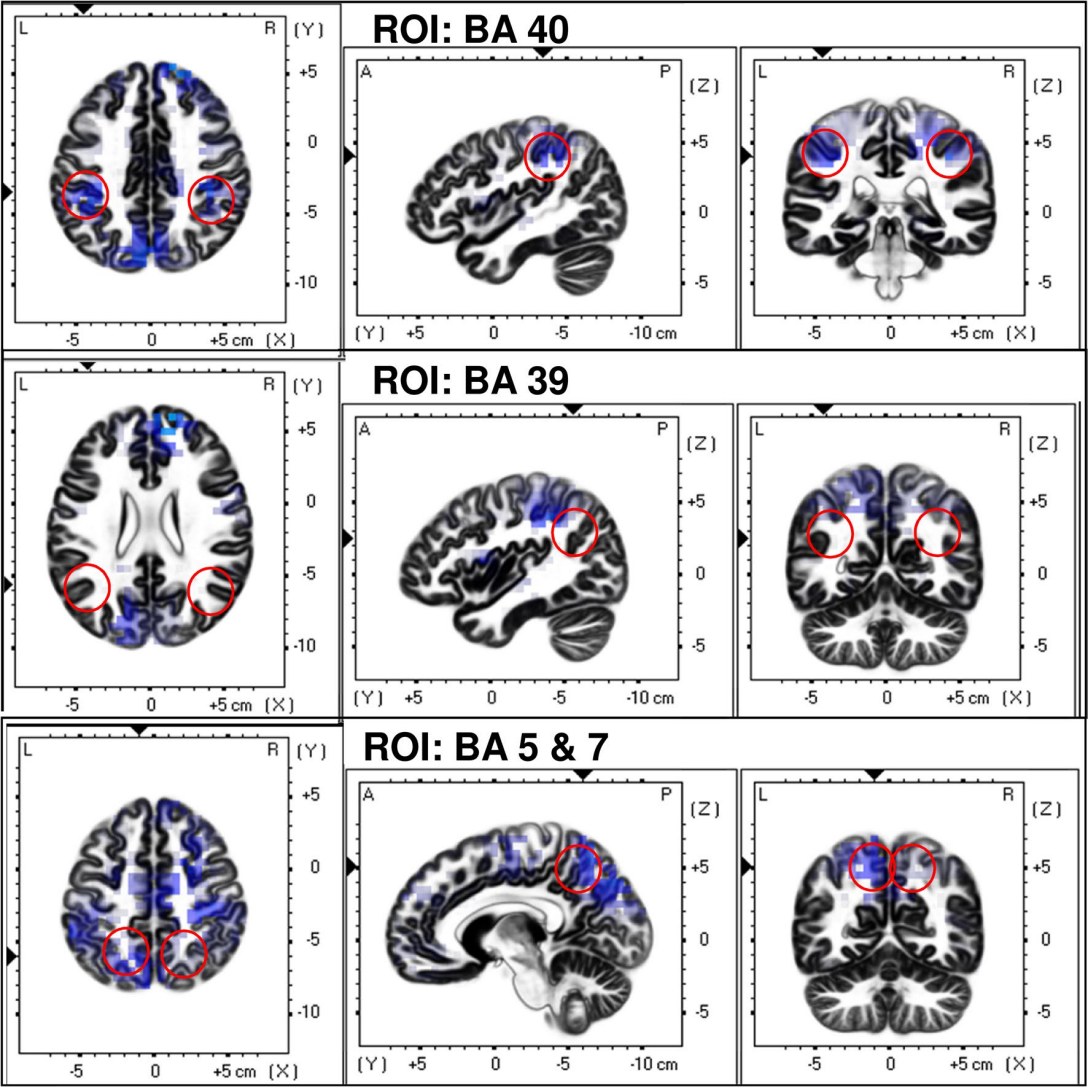
Comparison of brain activity for the P300 between patients with OCD and healthy controls by sLORETA. Here, the ROIs, namely BA39, BA4, BA 5 and BA7 are marked

For the analysis of source P300 data, sLORETA Software [57] was used. Therefore, the re-referencing was conducted to the average of all electrodes and the average of segments from target tones were exported. First, we compared the current density power, measured as µA/mm^2^, between groups. Therefore, a voxel-by-voxel *t*-test was performed on log-transformed data for the timeframe from 240 to 580 ms after target tone. As previously done, a non-parametric randomisation approach was applied [58] for correction for multiple comparisons. In addition, a ROI analysis was performed to investigate the electric neuronal activity as current source density power in the parietal cortices comprising all voxels of the Brodmann areas 5, 7, 39 and 40 (see Fig. 2). Here, Brodmann areas belonging to the posterior parietal cortex were selected, due to the involvement of this regions in higher-order functions [59]. Since we are interested in cognitive processing, as represented by P300, we chose the posterior parietal cortex and excluded anterior parietal cortical regions, which are also involved in somatosensory processes.

In this study, the BA 5-ROI covered a region extended in Talairach space from *x*: 0–40 and 0 to − 40, *y*: − 35 to − 50, *z*: 50–70 and included all voxels. The BA 7-ROI covered the region from *x*: 0 to − 40 and 40, *y*: − 50 to − 80, *z*: 30–70, also including all voxels. Similarly, BA 39 extended from *x*: − 35 to − 60 and 35–60, *y*: − 55 to − 80, *z*: 10–40 and BA 40 BA 39 from *x*: − 25 to − 65 and 25–65, *y*: − 20 to − 60, *z*: 15–60. The ROI analysis was done with the “ROI-Extractor” tool which averages the CSD values in the specified voxels. The brain model of LORETA is based on the Montreal Neurological Institute average MRI brain map (MNI 152), while the solution space is limited to the cortical grey matter, comprising 6239 voxels of 5-mm^3^ resolution. The mean source density power at each ROI within the time frame of 240−580 ms after target tone onset was computed for every participant. Finally, we calculated the average of all ROIs for each participant.

### Behavioural practice session of delay-discounting

We used a slightly modified version of an established decision-making paradigm described previously by Peters and Büchel [36]. Before scanning, all subjects completed an identical practice version of the task. The results of the pretest were used to adequately compute offers for the fMRI sessions and estimate the individual discounting rate *k*. The participants were ask to choose between a fixed immediate reward of €20 and higher but delayed rewards in 2, 7, 14, 28 or 40 days. The delayed rewards were computed individually for each participant to ensure that the delayed offer was chosen in approximately 50% of all trials. The amount of money at which the participants switched from accepting the immediate fixed reward to the delayed reward, also called the indifference amount, was calculated and converted into proportions of the fixed reward. Based on the hyperbolic function, these data were used to obtain the best-fitting discounting parameter *k*.

### fMRI

During fMRI, each trial began with a short cue symbol (500 ms) followed by presentation of the reward options (immediate vs. delayed) for 2000 ms. After a jittered anticipation period of 2000–3000 ms, participants had to choose the preferred reward option using an MR-compatible response box. After a short feedback period of 2000 ms, a jittered intertrial interval (3000–5000 ms) was presented. Each delay condition consisted of 14 trials, resulting in 70 trials per run. During 10 control condition trials, the participants were asked to choose either the left or right side of the screen without getting a reward. The experiment consisted of two runs of approximately 18 min each. Functional data were collected using a 3-T whole-body MRI system (Philips Achieva 3.0 T TX) equipped with a 32-channel Philips SENSE head coil. A total of 32 T2*-weighted echo-planar images per volume with blood-oxygen-level-dependent (BOLD) contrast were obtained using a sensitivity-encoded single-shot echo-planar imaging protocol (SENSE-sshEPI). For further details of fMRI procedures, see our previous publications [37, 60]. The functional data were preprocessed and statistically analysed using SPM8 (Wellcome Department of Cognitive Neuroscience, University College London, UK: http://www.fil.ion.ucl.ac.uk) and MATLAB 7.11 (Mathworks Inc., Natick, MA, USA). In addition to the whole brain analyses described elsewhere [37, 60], activity in anatomically defined regions of interest based on our previous work were analysed. These regions, namely the left and right OFC, respectively (inferior frontal gyrus, orbital part; superior frontal gyrus, medial orbital part (SFG/MO); middle frontal gyrus, orbital part; superior frontal gyrus, orbital part; gyrus rectus) were generated using both AAL and WFU PickAtlas software. More in detail, percent signal changes (based on the beta values for each event) derived from the above-mentioned regions were extracted using the standard routines implemented in MarsBar [61].

### Statistical analysis

Statistical analyses of the data were performed using IBM SPSS Statistics for Windows, Version 25.0 (IBM Corp., Armonk, NY, USA). The analyses of P300, questionnaire data and neuroimaging results were performed with nonparametric Mann–Whitney *U* tests and Spearman correlation coefficients due to violations of normal distribution. Statistical significance was defined as *p* < 0.05. For correlations of questionnaires and P300 data, Bonferroni correction due to multiple testing was applied, whereby related variables, e.g. P3 amplitude and P4 amplitude, were considered as one factor. The *p*-value threshold was shifted accordingly (for eight questionnaires: BDI, MOCI, STAI, CGI, MWST-IQ, NEO-FFI, BIS-11, PSP and three P300 variables: latency, amplitude and source density power: *p* = 0.05/11 = 0.0045). In the patients group, additional correlations were calculated for Y-BOCS scores. For correlations between fMRI data, based on ROI-analysis, and P300 (source P300 data), the significance level was set to *p* < 0.025 (since OFC regions are related and considered as one factor; correction for testing of left and right hemisphere was applied). For correlations between functional BOLD responses and P300, the significance threshold was adjusted for six different, unrelated regions and P300 (*p* = 0.05/7 = 0.007). The correlations with fMRI data were performed for the three different contrasts separately, i.e. [∆ immediate reward − control], [∆ delayed reward—control] and [∆ immediate reward—delayed reward].

## Results

### Sociodemographic and clinical findings

Patients with OCD reported significantly more severe psychopathological symptoms with higher scores in depression, anxiety and obsessive–compulsive symptom questionnaires compared to the control group (Table 1). Regarding personality characteristics, patients showed lower neuroticism (OCD: *M* = 1.31, SD = 0.69; control: *M* = 2.27, SD = 0.62; *U* = 48.0, *Z* = − 3.87, *p* < 0.001) and higher extraversion (OCD: *M* = 2.65, SD = 0.49; control: *M* = 2.05, SD = 0.60; *U* = 70.0, *Z* = − 3.23, *p* = 0.001) and openness to experience (OCD: *M* = 2.72, SD = 0.61; control: *M* = 2.32, SD = 0.48; *U* = 82.5, *Z* = − 2.86, *p* = 0.003). No differences between groups were observed for agreeableness and conscientiousness.

Although no differences between groups were observed for the BIS-11 total score, distinct differences emerged for the BIS-11 subscales, with OCD patients reaching lower scores in attentional impulsiveness (OCD: *M* = 12.58, SD = 3.08; control: *M* = 17.63, SD = 4.0; *U* = 42.5, *Z* = − 4.05, *p* < 0.001) and higher scores in motor impulsiveness compared to the control group (OCD: *M* = 21.47, SD = 2.59; control: *M* = 19.32, SD = 3.13; *U* = 104.0, *Z* = − 2.25, *p* = 0.025).

### EEG: P300 findings

**Fig. 3:**
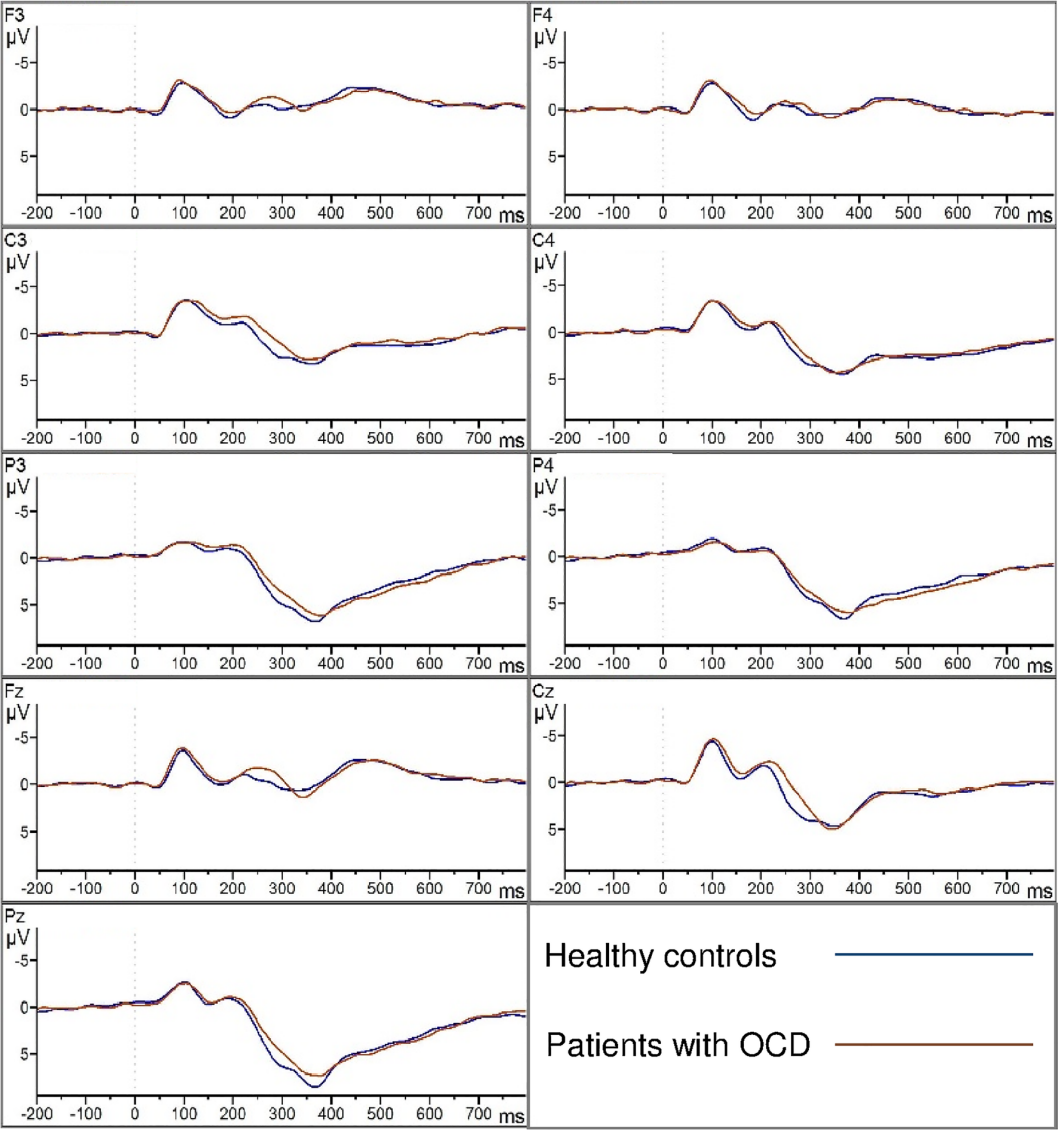
Grand-average waveforms showing the ERP components evoked by the target tones during the oddball paradigm. The waveforms of electrodes F3, F4 (first line), C3, C4 (second line), P3, P4 (third line), Fz, Cz (forth line) and Pz and the legend (sixth line) are presented. Healthy controls (blue) and patients with OCD (brown) are indicated by separate lines

The waveforms evoked by the target tones are shown in Fig. 3 for the parietal electrodes of interest (P3, P4 and Pz) and for additional central (C3, C4 and Cz) and frontal (F3, F4 and Fz) electrodes. Here, the parietal maximum of the P300 component is again observable. P300 amplitude and latency did not differ significantly between OCD patients and controls at P3, P4 and Pz. In OCD patients, amplitudes reached 8.5 µV (SD = 4.6), 7.0 µV (SD = 3.3) and 7.0 µV *(*SD = 3.5 µV) and latencies were 375.6 ms (SD = 53.0), 366.3 ms (SD = 50.8) and 373.7 ms (SD = 47.0) for Pz, P3 and P4, respectively. In healthy controls, amplitudes reached 8.5 µV (SD = 3.6), 6.9 µV (SD = 3.1) and 6.8 µV (SD = 2.9). There was a visual tendency towards shorter latencies within the control group (357.5 ms, SD = 28.5; 357.0 ms, SD = 23.5; 364.0 ms, SD = 36.9) for Pz, P3 and P4, respectively (see Fig. 3), compared to patients with OCD. This tendency is also visible in Figs. 1, which shows the parietal maximum in controls in the time window of 252–376 ms, and for patients with OCD, the most positive activity is observable in the last timeframe from 376 to 500 ms.

**Fig. 4:**
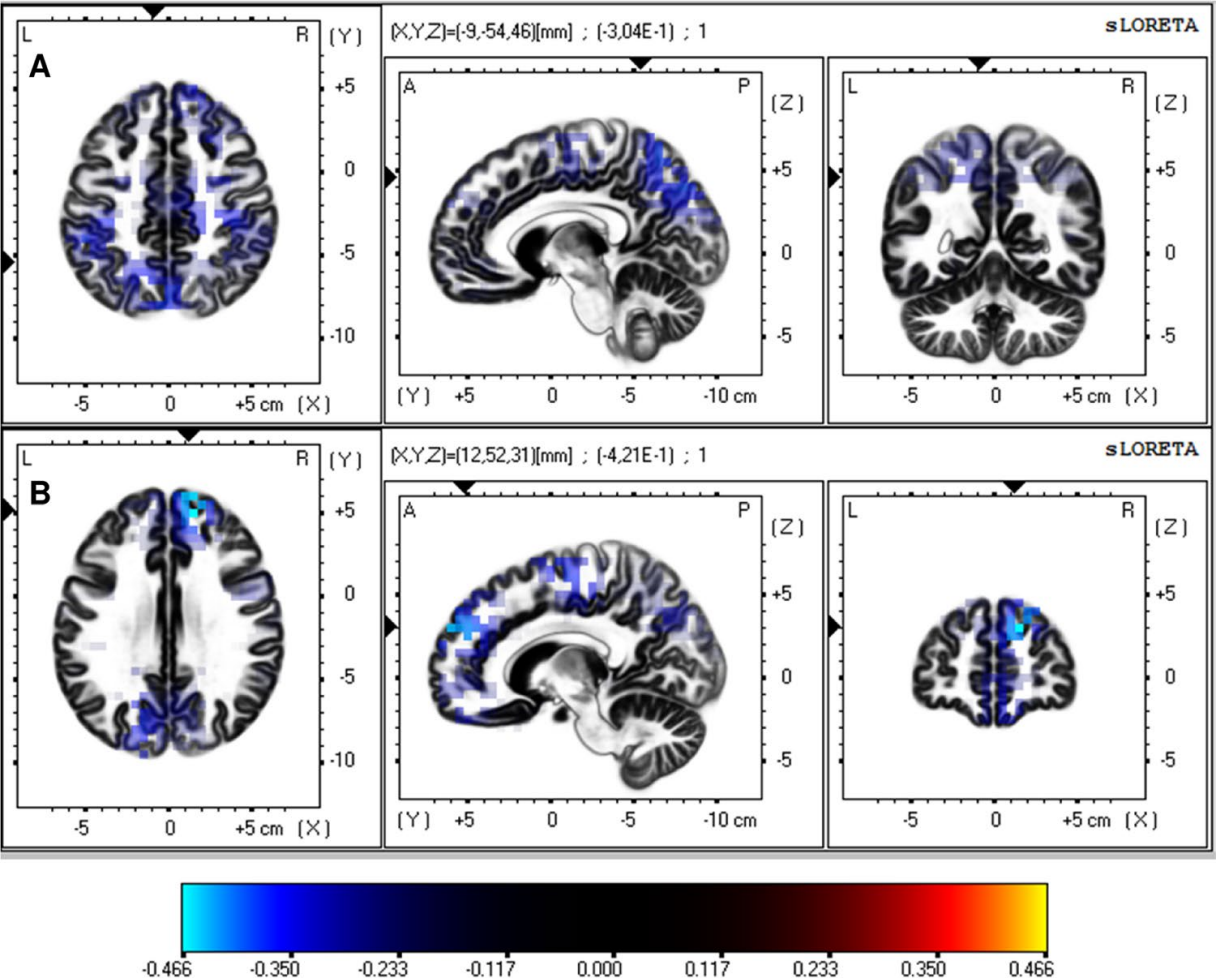
T-test comparison of current source density power by sLORETA between patients with OCD and healthy controls. The marked differences did not reach statistical significance. In **a**, parietal brain regions are shown and in **b** frontal regions are visible

Similar to the cortical P300 results, no differences between groups were found for source P300 results as calculated by sLORETA (maximum *t* = 2.419, *p* < 0.05; all *p*’s > 0.05; see Fig. 4). Accordingly, no differences were found for the ROI analyses (Fig. 2).

### Correlations between P300 (EEG) and clinical outcome

**Fig. 5:**
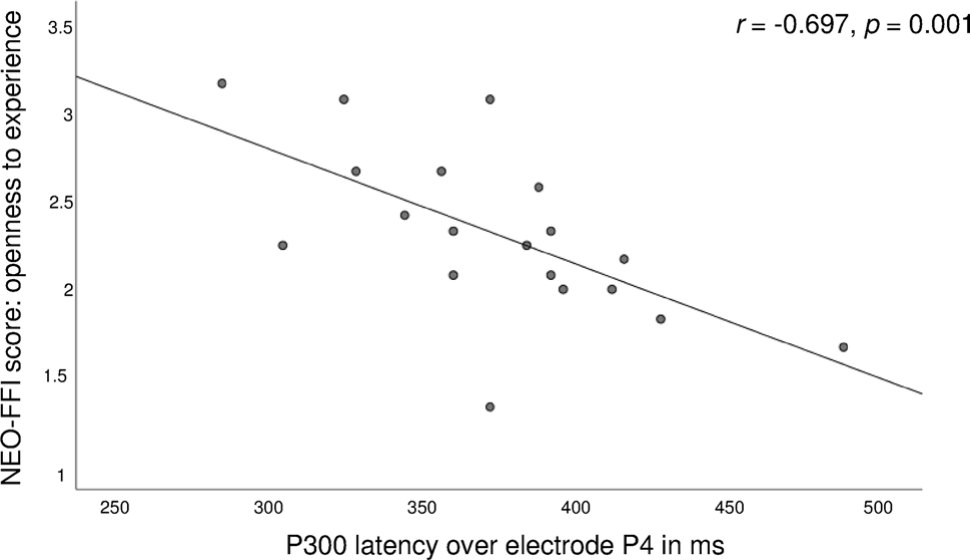
Correlation between the NEO-FFI score openness to experience score and the P300 latency over the P4 electrode in patients with OCD

In the group of healthy controls, questionnaire scores correlated with P300 characteristics, as measured by EEG. However, after correction for multiple testing (for nine questionnaires: BDI, MOCI, STAI, CGI, MWST-IQ, NEO-FFI, BIS-11, PSP and three P300 variables: latency, amplitude and source density power: *p* = 0.05/11 = 0.0045), no correlation remained significant. In the patients group, a significant correlation between the NEO-FFI openness to experience score and the P4 P300 latency survived Bonferroni correction (*r* = − 0.697, *p* = 0.001; see Fig. 5; correction for all variables mentioned above plus Y-BOCS).

### Correlations between P300 (EEG) and ROI-activation (BOLD)

**Fig. 6:**
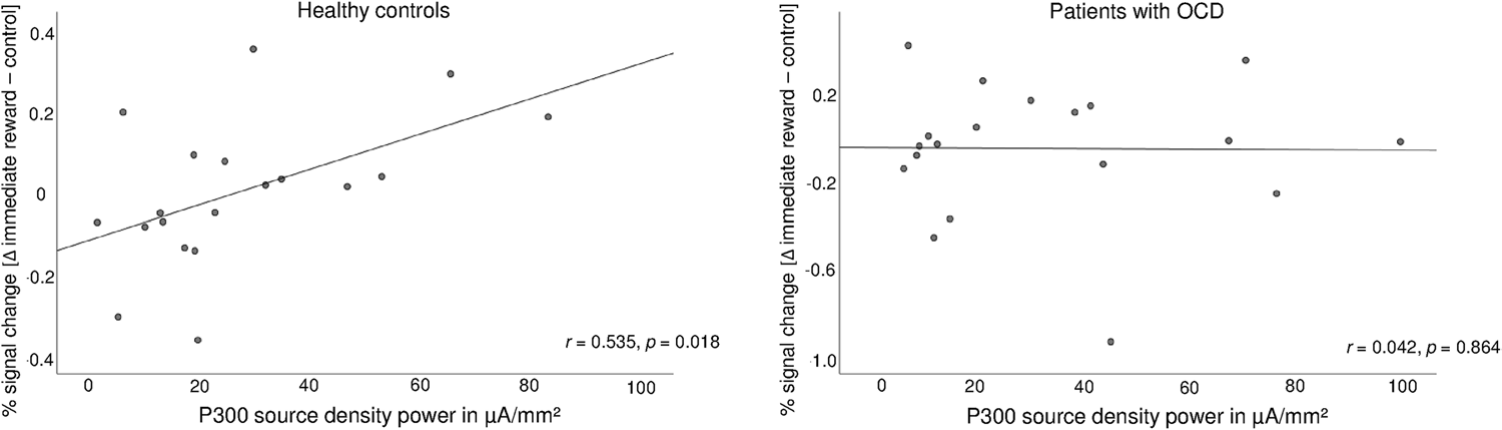
Correlations between activation in the left middle frontal gyrus (orbital part) for the difference [∆ immediate reward—control] and the P300 power over left parietal brain areas for healthy controls and patients with OCD

Regarding a possible relationship between reward-related neuronal activity during fMRI acquisition, extracted as signal change derived from anatomically based ROIs, and the P300 during EEG recording, we calculated the Spearman correlation coefficient for the fMRI signal for [∆ immediate reward—control] and P300 characteristics (source density power). Within the OCD group, no significant correlation was found. In contrast, we were able to detect significant positive correlations between activations, i.e. the signal change for the contrast, in the left middle frontal gyrus (orbital part) and P300 source density power (*r* = 0.535, *p* = 0.018; see Fig. 6) in the healthy subgroup. For the contrast [∆ delayed reward—control], significant correlations were again only observable in the control group between the signal change in the left middle frontal gyrus (orbital part) and the left superior frontal gyrus (orbital part) and the P300 source density power (*r* = 0.544, *p* = 0.016). For the contrast [∆ immediate reward—delayed reward], no significant association was found.

### fMRI BOLD responses and correlations with P300

For brain activations during the fMRI task in both groups, see Table 2 and for details see [37]. In brief, a main effect of task was observable in the bilateral inferior frontal gyrus, the bilateral supramarginal gyrus, the left middle frontal gyrus, the left middle occipital cortex and the angular gyrus. A group effect was observed for the left ventral striatum/putamen and the right dorsolateral prefrontal cortex. Correlations between these functional BOLD measures (FOI), based on the contrasts, and P300 source density power did not survive correction for multiple testing.

**Table 2:** Activations in healthy subjects and patients with obsessive–compulsive disorder (OCD)

	Hemisphere		Region	Extent *k*	*Z* value	Statistical value^a^
F-contrast [main effect of task] collapsed over groups						
− 38, 6, 28	L		Inferior frontal gyrus, opercular part	24	4.94	16.89
− 58, − 34, 34	L		Supramarginal gyrus	69	5.09	17.93
60, − 38, 34	R		Supramarginal gyrus	81	5.23	18.91
− 34, 28, 38	L		Middle frontal gyrus/dlPFC	31	4.88	16.51
56, 12, 8	R		Inferior frontal gyrus, opercular part^b^	16	3.51	9.06
− 30, − 76, 22	L		Middle occipital cortex^c^	50	4.24	12.64
28, − 54, 42	R		Angular gyrus^c^	12	3.95	11.13
T-contrast [Interaction group × task], i.e. “immediate reward: accepted” vs. “delayed reward: accepted” in heathy vs. OCD patients						
− 22, 16, − 2		L	Putamen/ventral striatum^b^	12	3.56	3.67
16, 20, 56		R	dlPFC (BA8)^b^	52	3.68	3.8

Initial threshold *p*[FWE] < 0.05 for an extent *k* > 10 voxels or *F* > 10.0 for *k* > 10

^a^*t* or *F* value. ^b^*p*[FWE] < 0.05 after small volume correction with 5 mm radius. ^c^*p*[FWE] < 0.05 on cluster level. *BA* Brodmann area, *dlPFC* dorsolateral prefrontal cortex

## Discussion

The present study investigated P300 ERPs and their associations with fMRI activation in a delay-discounting task in OCD patients and healthy controls. The two matched groups differed regarding psychopathology, personality characteristics and impulsivity but did not differ in P300 amplitudes or latencies or P300 source density power in parietal regions. Thus, our hypothesis that the groups will differ regarding P300 characteristics was not confirmed. Regarding personality characteristics, patients showed lower neuroticism, but higher extraversion and openness to experience. In previous studies, higher neuroticism and lower extraversion has been reported frequently for patients with OCD [62, 63]. Here, the findings also seem inconsistent, whereas it has been proposed by another study that facets of openness may impact on the particular expression and severity of obsessive–compulsive symptoms [64]. In our study, the factor openness was negatively correlated with P300 latency over P4 in the patients group (see Fig. 5). Thus, higher openness is related to smaller peak latencies, i.e. lower controlled processing. This could, very speculatively, interpreted as lower inhibition in individuals scoring high in openness to experience.

Even if patients with OCD did not differ from healthy controls with regard to P300 latencies and amplitudes, a tendency towards prolonged P300 latency was observed for OCD patients. Previous studies reported prolonged latencies and larger P300 amplitudes in OCD [8–14]. However, it should be noted that the existing literature on P300 EEG abnormalities in studies of patients with OCD is rather discrepant. Sanz et al. [17] found lower P300 amplitudes in combination with prolonged P300 latencies in a sample of drug-free adult OCD patients compared to healthy controls. In addition, a trend towards increased P300 amplitude was observed in patients after treatment with clomipramine, whereas, no modification in P300 latency was shown. Dayan-Riva and colleagues [65] utilised pictures showing neutral and angry facial expressions instead of auditory stimuli. They reported higher P3 amplitudes in patients with OCD compared to unaffected controls for neutral stimuli only, with no differences regarding angry facial expressions [65]. In this study, no differences were found between groups for latencies, suggesting that the different findings observed in OCD patients compared to healthy controls may depend crucially on the tasks used. In addition, it is known that P300 latencies have a much lower reliability than P300 amplitudes, whereas, perhaps data on P300 onset latency could have been mixed up with data on P300 peak latency. Most of these studies reported a shortened P300 latency whereas others detected prolonged latencies [9, 17]. In previous studies, shorter latencies in OCD patients were found only for P3b [8, 13, 14]. Thus, recent research brought several arguments for altered P300 amplitudes and more sparse support for latency differences in OCD. Regarding source analysis of P300, less research is existing, whereas, one study reported higher P300-related activity in patients with OCD in the left orbitofrontal cortex, left prefrontal, parietal and temporal areas compared to controls [13]. Thus, there are hints that altered P300 could play a role in OCD, whereas, the results may depend on the tasks used, the sample sizes investigated and medication of samples. Furthermore, the data analysis may have varied across studies, e.g. with regard to peak latency vs. onset latency analysis or the investigation of P3a and P3b subcomponents.

### P300 and delay-discounting in OCD

Previous researchers have revealed that P300 reflects the updating of cognitive models in order to make an appropriate response in the sense of an evaluation process for making a decision [22, 66]. In our fMRI study part, as previously reported for the present dataset, it has been shown that activations of dorsolateral PFC and ventral striatum activations differed between OCD patients and control participants during a delay-discounting paradigm (see [37]). Thus, it was known that P300 (context updating) during EEG recording and delay-discounting behaviour and processing were altered in OCD. Therefore, the question was whether P300, measured by EEG, is related to brain activations, measured by fMRI, during decision making, which was the secondary subject of the present study. In healthy participants, source density power of P300 over parietal brain areas correlated positively with activations in the left middle and superior frontal gyri (orbital parts) for the [∆reward—control] contrasts during the fMRI task. No such correlations were found in the patient’s group. The correlations in healthy controls are consistent with previous results, showing larger P300 amplitudes in contexts causing higher risk tendencies [21]. Furthermore, Bellebaum et al. [67] reported that P300 was larger for positive outcomes and showed an effect of potential reward magnitude that was independent of valence. Thus, findings regarding the relationship between P300 and decision-making suggested that P300 was modulated by reward magnitude.

This association was absent in patients with OCD, as no correlations of brain activation during the fMRI-task and P300 power density were found. There are several potential reasons for these findings. First, as we found lower scores for attentional impulsiveness in patients with OCD compared to healthy controls, a general reduced attention could attenuate the association of P300 with brain activation during the delay-discounting paradigm. Second, it has been suggested that patients with OCD exhibit prolonged deliberation during decision-making, implicating emotional valence or risk due to altered processing in relevant brain regions, including frontal and limbic regions [68]. Third, previous studies reported impaired adaption of the decision strategy during a decision-making task, suggesting lower flexibility in OCD [69, 70]. It can be speculated that the reduced flexibility could be related to reduced attention. In summary, previous research indicated decreased flexibility, and therefore, decreased capacity in OCD to focus attention in a goal-directed manner. In addition, deficits may occur due to delayed attention to relevant cues in OCD ([71]; for review, see [72]).

In fact, this interpretation is speculative and not based on our results. Based on our data, one can propose that these negative findings in the patient group could be caused by altered cognitive controlled processing in these patients, whereby controlled processing is not directly related to reward processing in the OFC, a region which is proposed to be hyperactive due to diminished inhibitory effects of the striatum in OCD. Altered activations of the dorsolateral PFC and the ventral striatum has been shown for the present group of patients, wherefore the results suggest that the OCD group showed indeed altered processing in cortico-striato-thalamo-cortical (CSTC) circuits during the fMRI-task. Therefore, the missing link between parietal cognitive processing, measured by EEG, and OFC activation during reward processing in the fMRI scanner in patients might reflect deviating CSTC circuit processing compared to processes observed in healthy individuals. Another possible reason for the missing association between general cognitive processing (EEG), and reward processing, measured by fMRI, in patients with OCD could be a diminishing effect of the psychopharmacological medication the patients received. In the present study, most of the patients received antidepressant medication. However, it has been shown that psychopharmacological medication affect P300 and OFC activity [73, 74]. Therefore, future research also might investigate the effect of psychopharmacological medication in cognitive processing. Finally, the sample sizes were small in the present study, wherefore significant results, also for P300 analyses between groups, would possibly appear in larger samples.

### Conclusion

In the present study, a negative correlation between the factor openness with P300 latency over P4 was observed exclusively in the patients group. We found distinct associations in healthy controls showing correlations of brain activation, as measured by fMRI during reward processing with P300 power, which were absent in the group of patients with OCD. Since cognitive processing, as indicated by P300, did not differ between the groups, the missing association in the group of patients with OCD could be interpreted as altered CSTC circuit activity, which would disrupt the association with general cognitive processing observed in unaffected individuals.

### Limitations

Some limitations of this current study should be noted. First, as mentioned above, our sample consists of patients receiving SSRI medication, which may have affected the results. Second, the small sample size does not enable a meaningful investigation of the specific OCD subgroup characteristics or maybe even group differences at all. Furthermore, P300 as well as fMRI BOLD contrasts during the delay-discounting task are both indirect measurements of brain activity. Finally, both measurements were recorded in sequence within a few hours, but not simultaneously, possibly producing a bias. Furthermore, the proportion of trait and state properties of P300 characteristics and brain activity during the delay-discounting task remains difficult to determine exactly.
